# Pathway Analysis Using Genome-Wide Association Study Data for Coronary Restenosis – A Potential Role for the PARVB Gene

**DOI:** 10.1371/journal.pone.0070676

**Published:** 2013-08-09

**Authors:** Jeffrey J. W. Verschuren, Stella Trompet, M. Lourdes Sampietro, Bastiaan T. Heijmans, Werner Koch, Adnan Kastrati, Jeanine J. Houwing-Duistermaat, P. Eline Slagboom, Paul H. A. Quax, J. Wouter Jukema

**Affiliations:** 1 Department of Cardiology, Leiden University Medical Center, Leiden, The Netherlands; 2 Department of Gerontology and Geriatrics, Leiden University Medical Center, Leiden, The Netherlands; 3 Department Human Genetics, Leiden University Medical Center, Leiden, The Netherlands; 4 Molecular Epidemiology, Leiden University Medical Center, Leiden, The Netherlands; 5 Deutsches Herzzentrum München, Technische Universität München, Munich, Germany; 6 Department of Medical Statistics and Bioinformatics, Leiden University Medical Center, Leiden, The Netherlands; 7 Netherlands Consortium for Healthy Ageing, Leiden University Medical Center, Leiden, The Netherlands; 8 Department of Vascular Surgery, Leiden University Medical Center, Leiden, The Netherlands; 9 Durrer Center for Cardiogenetic Research, Amsterdam, The Netherlands; 10 Interuniversity Cardiology Institute of the Netherlands (ICIN), Utrecht, The Netherlands; Yale School of Public Health, United States of America

## Abstract

**Background:**

Coronary restenosis after percutaneous coronary intervention (PCI) still remains a significant limitation of the procedure. The causative mechanisms of restenosis have not yet been fully identified. The goal of the current study was to perform gene-set analysis of biological pathways related to inflammation, proliferation, vascular function and transcriptional regulation on coronary restenosis to identify novel genes and pathways related to this condition.

**Methods:**

The GENetic DEterminants of Restenosis (GENDER) databank contains genotypic data of 556,099SNPs of 295 cases with restenosis and 571 matched controls. Fifty-four pathways, related to known restenosis-related processes, were selected. Gene-set analysis was performed using PLINK, GRASS and ALIGATOR software. Pathways with a p<0.01 were fine-mapped and significantly associated SNPs were analyzed in an independent replication cohort.

**Results:**

Six pathways (cell-extracellular matrix (ECM) interactions pathway, IL2 signaling pathway, IL6 signaling pathway, platelet derived growth factor pathway, vitamin D receptor pathway and the mitochondria pathway) were significantly associated in one or two of the software packages. Two SNPs in the cell-ECM interactions pathway were replicated in an independent restenosis cohort. No replication was obtained for the other pathways.

**Conclusion:**

With these results we demonstrate a potential role of the cell-ECM interactions pathway in the development of coronary restenosis. These findings contribute to the increasing knowledge of the genetic etiology of restenosis formation and could serve as a hypothesis-generating effort for further functional studies.

## Introduction

Although interventional cardiology has advanced tremendously in recent years, restenosis after percutaneous coronary intervention (PCI) still remains a significant limitation of the procedure. [1] Restenosis is a complex disease for which the causative mechanisms have not yet been fully identified. Genetic susceptibility is known to play a role in the development of this complication. [1] Many studies have focused to identify these genetic markers associated with restenosis. Candidate gene approaches investigating restenosis have resulted in identification of genetic variation in genes involved in the inflammatory response [2], [3] and genes related to vascular remodeling [4], [5] and proliferation [6]. In 2012, our group analyzed the joint effect of the genetic variation of 36 previously described candidate genes of restenosis, demonstrating that the joint effect of all the single nucleotide polymorphisms (SNPs) in these genes together was indeed significantly associated with restenosis development. [7] This association was mainly driven by SNPs in 6 individual genes, together spanning a wide range of different functions.

The first genome-wide association study (GWAS) on restenosis only identified one intergenic region on chromosome 12. [8] Although this finding, after further functional confirmation, potentially helps our understanding of the molecular background of restenosis, it also complicates things since none of the previously described markers were identified at a GWAS significant level. The main explanation for this is likely the multifactorial nature of the disease and thus the small individual effect size of the SNPs. It has been described that instead of examining individual SNPs, analyzing the joint effect of multiple SNPs is a better method to find genetic variation in complex diseases.[9]–[11] Incorporating biological knowledge of the interplay of several genes within functional pathways may improve the power to detect associated genes not previously identified for their involvement. Thus far, the reported genetic variants only explain a small part of the expected genetic component of restenosis development. [7] This so-called “missing heritability” is not uncommon in complex diseases, and pathway approaches are expected to fill at least part of this gap. [12], [13] During the last years, several studies have been reported that successfully applied this approach, for instance, identifying biological pathways related to human longevity [14], pancreatic cancer [15], crohn’s disease [16] and psychiatric disorders. [17], [18].

The goal of the current study was to examine the joint effect of genetic variation in biological pathways related to inflammation, proliferation, vascular function and transcriptional regulation on coronary restenosis in the GENetic DEterminants of Restenosis (GENDER) study population to identify novel genes and pathways related to this condition.

## Methods

### GENDER Study Population

The main results of the GENDER study [19] and the design and results of the genome-wide association study (GWAS) [8], [20], have been described previously. In brief, GENDER included 3,104 consecutive unrelated symptomatic patients treated successfully by PCI for angina. During a follow-up period of 9 months, the endpoint clinical restenosis, defined as renewed symptoms requiring target vessel revascularization (TVR) either by repeated PCI or CABG, by death from cardiac causes or myocardial infarction not attributable to another coronary event than the target vessel, was recorded. During follow-up, 346 patients developed clinical restenosis. Blood samples were collected at the index procedure for DNA isolation. The study protocol conforms to the Declaration of Helsinki and was approved by the ethics committees of each participating institution (the ethics committee of the Leiden University Medical Center, the ethics committee of the Academic Medical Center Amsterdam, the ethics committee of the University Medical Center Groningen and the ethics committee of the Maastricht University Medical Center). Written informed consent was obtained from each participant before the PCI procedure.

The GWAS was performed in a subpopulation of GENDER of 325 cases of restenosis and 630 controls matched by gender, age, and several other known risk factors for restenosis like total occlusion, diabetes, current smoking and residual stenosis. Genotyping was performed using the Illumina Human 610-Quad Beadchips following the manufacturer’s instructions. After genotyping, samples and genetic markers were subjected to a stringent quality control protocol. [20] The population substructure was analyzed by means of multidimensional scaling. The vast majority of the individuals fell in the same cluster along with the European population but 67 individuals were outside this cluster and were considered as genetic outliers and therefore excluded from further analyses. The final dataset consisted of 866 individuals (295 cases, 571 controls) and 556,099 SNPs that passed all quality control criteria, together covering 89% of the common genetic variation in the European population. [20].

### Pathway Selection

Pathways related to known restenosis-related processes (inflammation, vascular function, proliferation and transcription) were selected from the MSigDB database [21], which included curated gene sets from KEGG [22], BioCarta [23] and Reactome [24], [25]. A total of 54 pathways were selected (Table S1).

### Replication Cohort

The replication cohort consisted of a German restenosis cohort, i.e. patients presenting with ischemic symptoms or evidence of myocardial ischemia in the presence of ≥50% de novo stenosis located in native coronary vessels. All patients were treated with PCI and stent implantation at Deutsches Herzzentrum München or 1. Medizinische Klinik rechts der Isar der Technischen Universität München. The study protocol conformed to the Declaration of Helsinki and was approved by the institutional ethics committee responsible for both participating centers, Deutsches Herzzentrum München and 1. Medizinische Klinik, Klinikum rechts der Isar, Technische Universität München. All subjects gave their written, informed consent for participation in the study. The main characteristics of this cohort and the protocols of stent placement and post-stenting therapy have been described previously [26], [27]. Briefly, this population included 1537 patients treated with implantation of bare-metal stents (BMS) and 1920 patients treated with implantation of drug-eluting stents (DES). Target lesion revascularization (TLR) within 1 year after PCI was considered as the primary endpoint. Re-hospitalization for repeat angiography was scheduled between 6 and 8 months or earlier if non-invasive evaluation or clinical presentation suggested the presence of ischemia. For the current study we only analyzed the patients receiving BMS, since this was the only type of stent implanted during the GENDER study.

The samples during the replication stage were genotyped by means of iPLEX assays [28]. The SNPs were selected for replication after step-down fine-mapping of the significant associated pathways. Finally only SNPs within these pathways showing a p-value <0.10 and linkage disequilibrium (LD) <0.5 in the GENDER study were selected. Two assays were designed using MassArray design software (Sequenom, San Diego, CA). Genotyping was performed using iPLEX assays with use of the MassARRAY methodology (Sequenom), with alleles discriminated by mass spectrometry, following manufacturer’s instructions. One SNP, rs5764560, did not fit the assay. All the genotyped SNPs had a call rate >95%. One SNP, rs7292425, was out of Hardy Weinberg equilibrium (p-value<0.001), and was excluded for further analysis. Duplicate samples (∼2.5%) showed identical genotypes. Finally, 39 SNPs passed all quality criteria and were further analyzed.

### Statistical Analysis

Analyses were performed using PLINK [29], GRASS [30], and ALIGATOR [31] software. We will briefly describe all three methods. During the set-based test of PLINK the joint effect of all genetic variation, fulfilling the test constraints, within the set of genes of pathway of interest is evaluated. First a single SNP analysis of all SNPs within the pathway set is performed. Subsequently, a mean SNP statistic is calculated from the single SNP statistics of a maximum amount of independent SNPs with a p-value <0.2. SNPs are considered independent when the LD expressed in R^2^ is <0.5. Of the SNPs that are in LD with R^2^>0.5, the SNP with the lowest p-value in the single SNP analysis is selected. This analysis is repeated 10,000 times in simulated datasets with permutation of the phenotype. An empirical p-value for the SNP set is computed by calculating the number of times the test statistic of the simulated SNP sets exceeds that of the original SNP set.

GRASS calculates “eigenSNPs” for each gene in the pathway set by summarizing the variation of a gene using principal component analysis. Subsequently, one or more of these “eigenSNPs” per gene are selected using regularized logistic regression to calculate a test statistic for each pathway set. This analysis is repeated 10,000 times in simulated datasets with permutation of the phenotype. The p-value per pathway SNP set is calculated by comparing the test statistic of the original pathway SNP set with that of the combined simulated pathway SNP sets.

ALIGATOR (Association LIst Go AnnoTatOR) analyses gene sets for genes enriched with significant SNPs. Enrichment is defined as a gene set containing a larger number of significant genes than expected by chance. Replicate gene lists of the same length as the original are generated by randomly sampling SNPs (thus correcting for variable gene size). The lists are used to obtain p-values for enrichment for each gene set (by comparing the number of significant genes observed on the actual gene list to that observed on each replicate list), to correct these for testing multiple non-independent categories, and to test whether the number of significantly enriched categories is higher than expected. ALIGATOR uses data from all the SNPs tested in a gene and corrects for the variable numbers of SNPs per gene. Each gene is counted once regardless of how many significant SNPs it contains, thus eliminating the influence of LD between SNPs within genes. We used p-value cutoff <0.005 for SNPs, 5000 replicate gene lists and 1000 permutations as parameters to run ALIGATOR. Pathways were included when 2 or more individual genes contained significantly associated SNPs. [31], [32].

Considering the exploratory nature of the analysis and the considerate overlap between the pathways, associations of pathways with restenosis were considered worthwhile exploring with P<0.01. Since it has been suggested that PLINK, GRASS, and ALIGATOR provide complementary information [14], [33], we proceeded with the secondary analysis when a pathway was associated with restenosis with p<0.01 in at least one of the analyses. The pathways meeting this criterion were explored in more detail by fine-mapping of firstly the genes within those pathways and secondly the individual SNPs. For these secondary analyses, as well as during the replications stage p<0.05 was considered significant. When applying a strict Bonferroni correction, correcting for the 54 tested pathways and three different tests, the threshold for statistical significance was set at p<0.0003 ( = 0.05/(54*3).

## Results

The 295 patients with restenosis were well matched with the 571 patients without restenosis, for the known risk factors for restenosis, like age, diabetes, current smoking, stenting of the culprit lesion and previous restenosis (Table 1). Hypertension and multivessel disease were more common in the cases compared to the controls.

**Table 1:** Baseline characteristics of the GENDER study population.

	Cases (n = 295)	Controls (n = 571)	p-value
Age (years)	62.8±10.6	62.4±10.9	0.59
BMI (kg.m^−2^)	26.7±3.6	27.1±3.7	0.20
Male sex	213 (72)	421 (74)	0.63
Diabetes	58 (20)	119 (21)	0.68
Hypercholesterolemia	179 (61)	341 (60)	0.79
Hypertension	138 (47)	211 (37)	0.01
Current smoker	68 (23)	148 (26)	0.36
Family history of MI	117 (40)	210 (37)	0.41
Previous MI	119 (40)	246 (43)	0.44
Stable angina	188 (64)	400 (68)	0.06
Multivessel disease	155 (53)	248 (43)	0.01
Restenotic lesion	23 (8)	48 (8)	0.76
Total occlusion	57 (19)	97 (17)	0.40
Type C lesion	95 (38)	154 (27)	0.11
Stenting	199 (68)	385 (67)	0.99

Values were given as n (%) or mean ± SD. Diabetes was defined by treatment with anti-diabetic medication or insulin at study entry. Hypertension was defined as a blood pressure of either >160 mmHg systolic or >90 mmHg diastolic. Hypercholesterolaemia was defined as total cholesterol concentrations >5 mmol/L.

BMI: body mass index, MI: myocardial infarction. P-values are determined by Pearsons Chi-Square (discrete variables) or unpaired 2-sided t-test (continuous variables).

A total of 54 pathways were analyzed in the current study. Half of these pathways (n = 27) were related to inflammation, 13 pathways were related to proliferation, six pathways were related to vascular function and finally, eight pathways were related to transcriptional regulation. The pathways associated with restenosis with a p-value below 0.01 in either PLINK, GRASS or ALIGATOR are shown in Table 2. An overview of the results of all the pathways can be found in Table S1. When applying Bonferroni correction none of the pathways were significantly associated.

**Table 2:** Pathways associated with restenosis with PLINK or GRASS.

Pathway		Database	PLINK		GRASS	ALIGATOR
**Inflammation**						
	IL2 signaling pathway	BioCarta		**0.006**	0.012	0.08
	IL4 signaling pathway	BioCarta		0.02	0.04	**0.008**
	IL6 signaling pathway	BioCarta		**<0.001**	0.08	0.01
**Proliferation**						
	Role of Mitochondria in Apoptotic Signaling	BioCarta		0.80	**0.006**	**NS**
	PDGF Signaling Pathway	BioCarta		**0.008**	0.03	0.02
**Vascular function**						
	Cell-extracellular matrix interactions	Reactome		**0.009**	0.17	0.14
**Transcription**						
	Insulin signaling pathway	BioCarta		0.02	0.14	**0.001**
	Control of Gene Expression by Vitamin D Receptor	BioCarta		**0.008**	0.07	**0.003**

A complete overview of all pathways can be found in Supplementary Table S1. NS, not significant (pathway did not meet test criteria).

In the set-based analysis using PLINK, the IL6 signaling pathway described by BioCarta was associated most significantly with clinical restenosis with a p-value of 0.0008. Using the GRASS software, this pathway had a p-value of 0.08 and the ALIGATOR analysis resulted in p = 0.01. The strongest associated pathway using GRASS was the BioCarta mitochondria pathway with a p-value of 0.006. However, this pathway was not associated with restenosis using PLINK (p = 0.80) or ALIGATOR. The insulin signaling pathway, described by BioCarta, was the strongest associated pathway using ALIGATOR (p = 0.001). Using PLINK this pathway had a p-value of 0.02, whereas GRASS resulted in p = 0.14. Other pathways that were associated in one of the analyses, considering the p<0.01 cut-off, include the inflammatory IL2 signaling pathway (PLINK p = 0.006, GRASS p = 0.012 and ALIGATOR p = 0.08) and the IL4 signaling pathway (PLINK p = 0.02, GRASS p = 0.04 and ALIGATOR p = 0.008, the proliferation platelet derived growth factor (PDGF) pathway (PLINK p = 0.008, GRASS p = 0.03 and ALIGATOR p = 0.02) and a transcriptional regulatory pathway, the vitamin D receptor (VDR) pathway (PLINK p = 0.008, GRASS p = 0.07 and ALIGATOR p = 0.003). Finally, the cell-extracellular (ECM) matrix interactions pathway, as described by Reactome, was demonstrated to be associated in PLINK (p = 0.009), but not in GRASS (p = 0.17) or ALIGATOR (p = 0.14).

Subsequently, fine mapping analysis was performed of six pathways with a p-value <0.01 in one of the three analyses. The individual genes of each pathway were analyzed using set-based analysis of PLINK (Table S2). Five of the 22 genes of the IL6 signaling pathway were significantly associated, as shown in Table 3. These genes were the RAF1 gene with a p-value of 0.001, the JAK2 gene with p = 0.006, the STAT3 gene with p = 0.006, the IL6 gene with p = 0.04, and the GRB2 gene with a p-value of 0.04 (Table 3). The IL2 signaling pathway and the PDGF signaling pathway showed considerable overlap with the IL6 pathway. Thirteen of the 22 genes (59%) of the IL2 pathway and 14 of the 32 genes (44%) of the PDGF pathway are also involved in the IL6 pathway. In addition to RAF1, JAK2 and GRB2, mentioned above, STAT5A, STAT5B and PIK3R1 were also significantly associated, p = 0.04, p = 0.01 and p = 0.04 respectively. The association of the VDR pathway was driven by three genes, NCOR1 (p = 0.003), VDR (p = 0.01) and BAZ1B (p = 0.02). The association of the cell-ECM interactions pathway was caused by two genes. The PARVB gene was associated with restenosis with a p-value of 0.001 and the LIMS2 gene with a p-value of 0.01. Finally, DIABLO was the only associated gene of the mitochondria pathway with a p-value of 0.03.

**Table 3:** Fine mapping of the associated pathways and replication of the top SNP.

						GENDER (N = 866)					Replication cohort (N = 1537)			
			SNPs	SNPs	P		MAF			P	MAF			P
Pathway	Gene	HGNC ID	(total)	(final)	(gene)	SNP	case	control	OR	(SNP)	case	control	OR	(SNP)
**IL6 signaling pathway**														
	RAF1	9829	19	2	0.001	rs1532534	23%	16%	1.58	<0.001	19%	18%	1.07	0.59
						rs1532533	46%	41%	1.26	0.02	37%	39%	0.91	0.34
	JAK1	6190	30	8	0.006	rs2256298	20%	28%	0.64	<0.001	**23%**	**27%**	**0.81**	**0.06**
						rs310219	22%	30%	0.66	<0.001	25%	27%	0.88	0.23
						rs2780900	8%	12%	0.61	0.005	11%	12%	0.94	0.69
						rs17127169	7%	11%	0.62	0.01	7%	8%	0.90	0.56
						rs310247	41%	47%	0.79	0.02	**40%**	**44%**	**0.84**	**0.07**
						rs974019	11%	14%	0.76	0.09	c			
						rs3790541	15%	12%	1.28	0.09	11%	10%	1.05	0.76
						rs12745769	22%	20%	1.19	0.16	d			
	STAT3	11364	7	1	0.006	rs6503695	42%	34%	1.35	0.004	35%	36%	0.95	0.61
	IL6	6018	10	1	0.04	rs11766273	8%	5%	1.58	0.02	7%	8%	0.87	0.43
	GRB2	4566	11	3	0.04	rs12950752	29%	22%	1.41	0.003	25%	26%	0.93	0.53
						rs16967789	17%	13%	1.35	0.03	15%	14%	1.01	0.94
						rs930297	13%	11%	1.28	0.11				
**IL2 signaling pathway**														
	RAF1	9829	19	2	<0.001	a								
	JAK1	6190	30	8	0.006	a								
	STAT5B	11367	5	1	0.01	rs8064638	38%	31%	1.33	0.007	31%	31%	0.97	0.77
	GRB2	4566	11	3	0.04	a								
	STAT5A	11366	3	1	0.04	rs8072785	13%	9%	1.44	0.02	11%	10%	1.16	0.32
**PDGF Signaling pathway**														
	RAF1	9829	19	2	0.001	a								
	STAT3	11364	7	1	0.006	a								
	JAK1	6190	30	8	0.006	a								
	STAT5B	11367	5	1	0.01	b								
	PIK3R1	8979	31	5	0.04	rs17318918	16%	11%	1.58	0.002	10%	9%	1.20	0.25
						rs10515074	27%	22%	1.31	0.02	e			
						rs1043526	17%	13%	1.36	0.03	13%	13%	1.00	0.99
						rs4122269	32%	37%	0.82	0.06	35%	36%	0.98	0.80
						rs40419	40%	43%	0.87	0.19	d			
	STAT5A	11366	3	1	0.04	b								
	GRB2	4566	11	3	0.04	a								
**VDR pathway**														
	NCOR1	7672	14	1	0.003	rs3744328	4%	1%	2.73	0.002	3%	3%	0.99	0.96
	VDR	12679	33	4	0.01	rs11574027	4%	1%	4.19	<0.001	2%	1%	1.08	0.83
						rs11574077	7%	4%	1.60	0.03	4%	5%	0.85	0.47
						rs11168287	42%	47%	0.83	0.07	48%	48%	0.96	0.70
						rs9729	46%	50%	0.88	0.19	d			
	BAZ1B(WSTF)	961	6	1	0.02	rs17400042	3%	6%	0.49	0.009	2%	3%	0.71	0.30
**Cell-extracellular matrix interactions pathway**														
	PARVB	14653	93	24	0.001	rs139107	16%	9%	1.91	<0.001	12%	13%	0.97	0.81
						rs2092351	31%	40%	0.67	<0.001	37%	37%	1.01	0.89
						rs6006636	18%	12%	1.53	0.002	15%	16%	0.90	0.42
						rs2179559	18%	24%	0.70	0.01	21%	19%	1.13	0.29
						rs2267617	52%	45%	1.33	0.01	49%	49%	1.03	0.72
						rs139069	23%	17%	1.41	0.01	20%	18%	1.14	0.24
						rs2072949	8%	5%	1.72	0.01	7%	6%	1.12	0.54
						rs2267604	28%	23%	1.34	0.01	f			
						rs139096	31%	25%	1.33	0.01	27%	29%	0.88	0.21
						rs5764455	39%	44%	0.80	0.03	**36%**	**42%**	**0.77**	**0.007**
						rs9614207	33%	38%	0.80	0.04	39%	37%	1.10	0.31
						rs6006611	50%	45%	1.23	0.04	48%	46%	1.12	0.23
						rs5764066	22%	18%	1.27	0.05	**26%**	**22%**	**1.23**	**0.05**
						rs2092437	14%	11%	1.33	0.07	15%	13%	1.20	0.18
						rs7292425	26%	22%	1.24	0.07	g			
						rs133911	26%	30%	0.81	0.07	30%	29%	1.05	0.65
						rs2073080	17%	20%	0.78	0.07	16%	17%	0.91	0.45
						rs5764560	41%	36%	1.20	0.08	h			
						rs8140244	13%	16%	0.81	0.14	d			
						rs5764495	47%	51%	0.87	0.16	d			
						rs2896022	46%	50%	0.87	0.17	d			
						rs2281292	38%	41%	0.87	0.19	d			
						rs6006615	19%	16%	1.19	0.19	d			
						rs6006486	28%	25%	1.16	0.19	d			
	LIMS2	16084	14	2	0.01	rs2288657	5%	2%	2.52	<0.001	4%	5%	0.90	0.65
						rs2243934	6%	4%	1.38	0.17	d			
**Mitochondria pathway**														
	DIABLO	21528	2	1	0.03	rs12870	48%	42%	1.27	0.02	44%	43%	1.03	0.72

In the first five columns the analysis of the separate genes is depicted. In the second five columns the single SNP analysis is shown and in the final four columns the replication stage is shown.

SNPs (total) indicates the total number of available SNPs in the gene. SNPs (final) indicates the number of SNPs with P<0.2 and LD<0.5 that were included in the set-based analysis. MAF, minor allele frequency. HGNC ID, HUGO Gene Nomenclature Committee unique identification number.

a single SNP analysis shown in IL6 pathway.

b single SNP analysis shown in IL2 pathway.

c SNP in LD (R^2^ = 0.52) with rs310219, not selected for replication.

d p-value>0.1 of SNP in GENDER, not selected for replication.

e SNP in LD (R^2^ = 0.57) with rs17318918, not selected for replication.

f SNP in LD (R^2^ = 0.54) with rs6006636, not selected for replication.

g SNP out of HWE (p = 0.0001) during replication.

h SNP did not fit the iPlex designs.

In Table 3 the results of the single SNP analysis of the significantly associated genes is reported showing which SNPs are responsible for the significant association of these genes. Of the in total 54 individual SNPs, 34 were associated with restenosis with a p-value below 0.05. The strongest associated SNP was located in the PARVB gene of the cell-ECM interactions pathway (rs139107, p<0.001).

Baseline characteristics of the replication cohort can be found in Table S3. In the replication stage, two SNPs in the PARVB gene, which was part of the cell-ECM interactions pathway, were the only SNPs found to be significantly associated with restenosis (Table 3). The minor allele of rs5764455 was significantly more prevalent in the control group than in the cases, 42% and 36% respectively, p = 0.007. In GENDER the minor allele frequency (MAF) of this SNP was 44% and 39% in controls and cases respectively, p = 0.03. For rs5764066 the MAF was 22% in controls and 26% in cases, p = 0.05, compared to 18% versus 22% in GENDER. In addition, two SNPs in the JAK1 gene, which was part of the IL6 pathway, the IL2 pathway and the PDGF pathway, showed a borderline significant association with target lesion revascularization in the replication cohort. No wet-lab experiments were performed to elucidate the function of the two PARVB SNPs, rs5764455 and rs5764066, but online databases were explored for available data. Both SNPs were located in introns and the UCSC genome browsers database (http://genome.ucsc.edu) did not indicate that they were located in a DNAse I hypersensitive site or in other important regulatory regions. Moreover, neither of two SNPs were reported in two publically available eQTL databases (VarySysDB [34] and the eQTL database of the Pritchard lab [35]).

## Discussion

We explored the joint effect of genetic variation in biological pathways on coronary restenosis development. When using a strict Bonferroni correction, none of the pathways reached the threshold of significance. However, we show a potential role for the cell-ECM interactions pathway as described by Reactome. This pathway, related to vascular remodeling, was associated with coronary restenosis in the GENDER study, in particular the PARVB gene and several SNPs in this gene were replicated in an independent study cohort. The SNPs of the five other pathways, associated in the GENDER study, did not replicate.

ECM remodeling and vascular smooth muscle cell migration and proliferation are thought to be the key processes in restenosis development. [1] Cell-ECM interactions, as described by the cell-ECM interactions pathway, play a critical role in regulating a variety of cellular processes in multicellular organisms including motility, shape change, survival, proliferation and differentiation. The PARVB (beta-parvin, also known as affixin, Gene ID: 29780) in particular, encodes a member of the parvin family of actin-binding proteins, which play a role in cytoskeleton organization and cell adhesion. Unlike other family members like alpha-parvin and gamma-parvin, beta-parvin has been shown to be expressed the highest in heart and skeletal muscle tissue. [36] PARVB is known to interact with integrin-linked kinase (ILK) and this complex is involved in the early stage of cell-substrate interaction through integrins. [37] Moreover, it has been suggested that it is a crucial modulator of cell survival [38] and that it has an important role in the angiogenic process [39], making it a likely candidate for restenosis development.

Since the introduction of GWAS many new insights on a wide array of disease have been reported. [40] However, extending these findings to biological mechanisms that explain disease development is a major ongoing challenge. The most likely explanation for this problem is that GWAS only focuses on single SNP associations. The reported loci often are located in intergenic regions or gene deserts and often are of unknown function. Furthermore, these loci have exhibited only modest effect sizes. Since biological pathways are the natural causative mechanisms resulting in disease and it is conceivable that genes functioning within these pathways interact within biological networks, it is only logical that the next step in the post-GWAS era is the exploration of these pathways. The rationale of set-based analysis is to overcome the marginally weak effect of single SNPs by analyzing a set of SNPs, since these SNPs could jointly have strong genetic effects. Examination of genetic variation of complete biological pathways, like in this set-based analysis, is therefore more likely to detect an associated mechanism by combining multiple SNPs with a possible marginal individual effect. [41], [42] In this study, the SNP with the strongest association with restenosis in the GENDER population was located in the PARVB gene of the cell-ECM interactions pathway (rs139107, p<0.001). As a reference, none of the SNPs currently examined, were located in the top 100 strongest associated SNPs of the complete GWAS analysis.(8).

For the current study we performed the analyses with PLINK, GRASS and ALIGATOR software. To date, many different statistical programs have been described to perform this type of analysis with. All methods have advantages and disadvantages. [9] In the case of PLINK and GRASS, both methods use the raw genotypes as input data. An advantage of this method is that they can take into account the LD structure within genes during their computations, which is not the case with methods performing their analyses using p-values as input data. Furthermore they both are self-contained tests that deal with the complexity of the SNP sets by permutation of the case-control status. However, differences between PLINK and GRASS include that PLINK analyzes SNP sets as a whole, and does not take the SNP distribution over the genes within the pathway into account, whereas GRASS first calculates “eigenSNPs” for each gene in the pathway set by summarizing the variation of a gene using principal component analysis. Compared to PLINK and GRASS, ALIGATOR is not a self-contained test but a competitive test, using SNP p-values instead of raw-genotypes, aiming to test whether genes in one pathway are enriched with a greater number of associated SNPs. Several comparative analyses have been performed, not resulting in a consensus of the best method, but rather suggesting to use two or more algorithms resulting in complementary findings. [14], [33].

For the current study we analyzed the data using a p-value threshold for including individual SNPs in the PLINK analyses of p<0.2 for their association with restenosis in the primary single SNP analysis. A standard setting in PLINK is often p<0.05, on relatively arbitrary grounds. Since we aimed at analyzing the combined effect of SNPs with small individual effect sizes we decided to increase this threshold to p<0.2. By increasing this threshold, more SNPs will be selected for further analyses ensuring that also the SNPs that were not associated with restenosis on their own, but that potentially did contribute to the overall effect of a pathway were taken into account. Before the start of this study we decided to apply a threshold of p<0.01 for the association of a pathway with restenosis, to identify pathways of interest. Although this threshold could be considered arbitrary, we decided that is was appropriate considering the considerate overlap between the pathways and the exploratory nature of this study. When using a strict Bonferroni correction for the 54 analyzed pathways and 3 applied tests (0.05/(54*3) = p<0.0003), none of the pathways would have qualified for further investigation. This could indicate that either the population is too small to detect the subtle genetic influence on this complex phenotype or that no solid genetic association is present. However, since to our knowledge, other (larger) restenosis study populations with GWAS data do not exist, we are limited in exploring this hypothesis regarding the sample size issue. A more strict multiple testing correction than currently used increases the risk of false negative results. Future functional studies are needed to further elucidate the biological background of restenosis development. The first step of the functional analyses would likely be to see whether the SNPs affect expression or function of the gene they are located in. If this is not the case, they could influence on other (nearby) genes, which could for instance be explored by mRNA expression analyses or allelic expression imbalance. [43] Furthermore, sequencing of the genes of interest could result in identifying functional variants in LD with the SNPs identified in the current study.

### Limitations

Proper replication of these analyses requires other datasets with GWAS data. To our knowledge, the GWAS performed in the GENDER study population is the first and unfortunately is currently the only GWAS on coronary restenosis. The desired replication cohorts are therefore unavailable. Since we did want to analyze our findings in an independent study cohort we decided to genotype only the top SNPs of the associated pathways instead. Considering the minimal replication of the analyzed SNPs, the robustness of the reported associations is limited. However, the presented data do provide evidence that ECM interactions and inflammation contribute to coronary restenosis development. And the results of the current study can serve as a hypothesis-generating effort for further functional studies.

All patients in the GENDER study and in the replication cohort were either treated with balloon-angioplasty (33% in GENDER) or BMS implantation. It is unknown whether these findings are generalizable to patients treated with DES or endothelial-progenitor-cell covered stents.

### Conclusion

In conclusion, although these results do not comply to the required strict multiple testing correction, we do demonstrate a potential role of the cell-ECM interactions pathway in the development of coronary restenosis. After performing additional studies, these findings could contribute to the increasing knowledge of the genetic etiology of restenosis formation. Unraveling the mechanisms leading to restenosis development remains challenging. There is a need for larger study populations specifically designed for genetic association analyses. In the select population that will still develop coronary restenosis despite advances in therapy, it will remain an imported cause of morbidity and therefore further understanding of the condition will be essential if we want to achieve personalized treatment and eradicate this costly and clinically very relevant limitation of PCI.

## Supporting Information

Table S1 Pathway analysis of pathways potentially related to restenosis.
(XLS)

Table S2 SNP set analysis of all the separate genes of the top pathways.
(XLS)

Table S3 Baseline characteristics of German BMS cohort (N = 1537).
(XLS)
